# Supplementary material for: The Role of Cytokines Produced via the NLRP3 Inflammasome in Mouse Macrophages Stimulated with Dental Calculus in Osteoclastogenesis
Source: Int J Mol Sci. 2021 Nov 18;22(22):12434. doi: 10.3390/ijms222212434 (PMC8618367; doi:10.3390/ijms222212434)
Supplement: Supplementary file 1 [file ijms-22-12434-s001.zip › ijms-1445735-supplementary.pptx]

## Slide 1
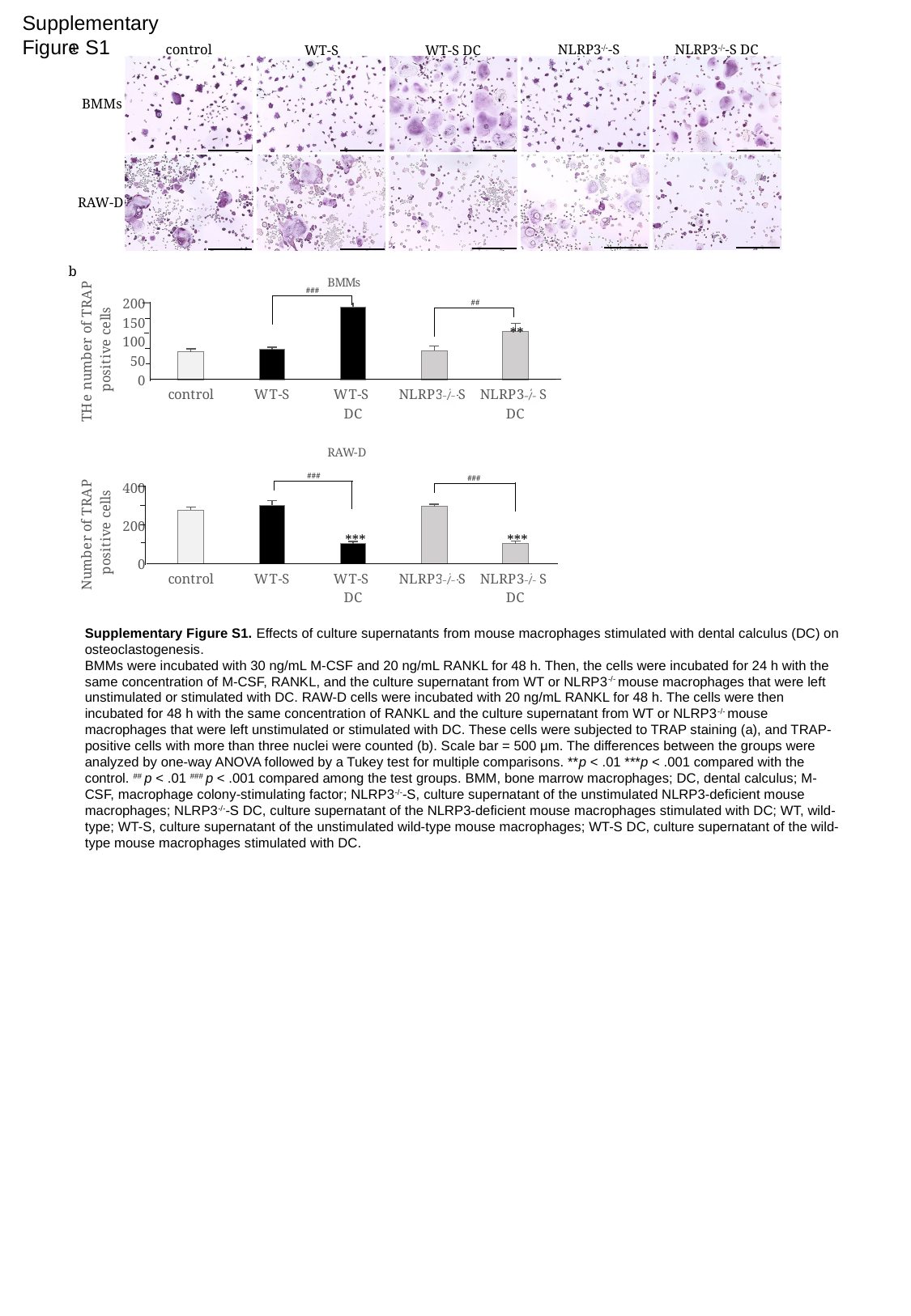

Supplementary Figure S1
control
NLRP3-/--S DC
NLRP3-/--S
WT-S DC
WT-S
BMMs
RAW-D
### Chart: BMMs
| Category | |
|---|---|
| control | 73.33333333333333 |
| WT-S | 79.66666666666667 |
| WT-S
DC | 189.33333333333334 |
| NLRP3-/--S | 76.33333333333333 |
| NLRP3-/--S
DC | 127.0 |###
##
***
**
### Chart: RAW-D
| Category | |
|---|---|
| control | 282.6666666666667 |
| WT-S | 308.3333333333333 |
| WT-S
DC | 105.66666666666667 |
| NLRP3-/--S | 300.6666666666667 |
| NLRP3-/--S
DC | 104.66666666666667 |###
###
***
***
a
b
Supplementary Figure S1. Effects of culture supernatants from mouse macrophages stimulated with dental calculus (DC) on osteoclastogenesis.
BMMs were incubated with 30 ng/mL M-CSF and 20 ng/mL RANKL for 48 h. Then, the cells were incubated for 24 h with the same concentration of M-CSF, RANKL, and the culture supernatant from WT or NLRP3-/- mouse macrophages that were left unstimulated or stimulated with DC. RAW-D cells were incubated with 20 ng/mL RANKL for 48 h. The cells were then incubated for 48 h with the same concentration of RANKL and the culture supernatant from WT or NLRP3-/- mouse macrophages that were left unstimulated or stimulated with DC. These cells were subjected to TRAP staining (a), and TRAP-positive cells with more than three nuclei were counted (b). Scale bar = 500 μm. The differences between the groups were analyzed by one-way ANOVA followed by a Tukey test for multiple comparisons. **p < .01 ***p < .001 compared with the control. ## p < .01 ### p < .001 compared among the test groups. BMM, bone marrow macrophages; DC, dental calculus; M-CSF, macrophage colony-stimulating factor; NLRP3-/--S, culture supernatant of the unstimulated NLRP3-deficient mouse macrophages; NLRP3-/--S DC, culture supernatant of the NLRP3-deficient mouse macrophages stimulated with DC; WT, wild-type; WT-S, culture supernatant of the unstimulated wild-type mouse macrophages; WT-S DC, culture supernatant of the wild-type mouse macrophages stimulated with DC.
